# Jumping Genes Cross Plant Species Boundaries

**DOI:** 10.1371/journal.pbio.0040035

**Published:** 2005-12-20

In the early 1950s, legendary plant geneticist Barbara McClintock found the first evidence that genetic material can jump from one place to another within the genome. The variegated kernels of her maize plants, she determined, resulted from mobile elements that had inserted themselves into pigment-coding genes, changing their expression. McClintock's mobile elements, or transposons, moved over generations within a single species. More recently, another form of genetic mobility has been discovered—genetic information can sometimes be transferred between species, a process called horizontal gene transfer. While horizontal genetic transfer occurs most commonly in bacteria, it has been detected in animals as well. Most transfers between higher animals involve the movement of transposons. Horizontal transfer can also occur between the mitochondrial DNA of different plant species. Until now, however, no one had found evidence for horizontal transfer in the nuclear DNA of plants.

In a new report, Xianmin Diao, Michael Freeling, and Damon Lisch studied the genomes of millet and rice, two distantly related grasses that diverged 30–60 million years ago. While the two grasses show significant genetic divergence from accumulating millions of years of mutations, they carry some transposon-related DNA segments that are surprisingly similar. The authors conclude that these sequences were transferred horizontally between the two plants long after they went their separate ways.

Transposons of the class identified by Diao et al. typically consist of a variable length of DNA that codes for one or more enzymes flanked by repeating sequences called terminal inverted repeats (TIRs). These repeats can bind to each other to form a “lollipop” that is easily excised from the DNA strand, carrying the rest of the transposon along with it. Plant genomes are rife with transposons, many of which are relatively passive. Transposons from the “Mutator” family in maize, however, are especially active, frequently causing mutations as they insert themselves into new positions in the genome. They perform this jump with assistance from the two proteins they code for, a transposase and a helper gene.

DNA from many species of plants contains several families of cousins of the Mutator transposons. These “Mutator-like elements,” or MULEs, code for a protein similar to the transposase, as well as the TIR sequences. Diao et al. identified 19 distinct MULEs in the DNA of various species of millet (genus *Setaria*), and compared these with the rice genome sequence, which was published in 2002. They compared the sequence similarity of these MULEs to that of other proteins that are also conserved in the same species for which sequences are available. Strikingly, they observed much higher sequence similarity between the MULEs from millet and rice than is typical for transposons. The greater similarity of the MULE DNA is easily explained if it jumped somehow, horizontally, between the species, but there could be alternative explanations. The match could have arisen without horizontal transfer, for example, if the MULE DNA had been under positive selection, as typically happens for protein-coding genes that confer some survival or reproductive benefit. In such cases, natural selection tends to preserve the integrity of these sequences. [Fig pbio-0040035-g001]

**Figure pbio-0040035-g001:**
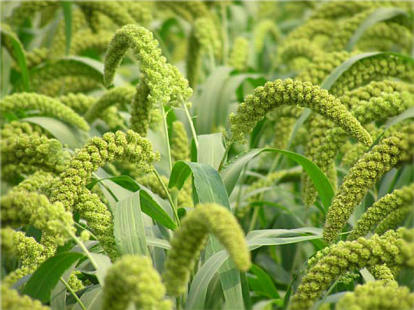
A genome-wide analysis of millet (above) and rice revealed the first clear evidence of horizontal gene transfer in plants

To test for signs of selection, the researchers looked at regions of the MULE DNA that don't appear to code for protein. The similarity between these noncoding regions in millet and rice MULEs was just as high as for the coding regions, even though selection probably doesn't influence them. Even within the coding sections, “synonymous” mutations—which don't change the protein sequence and so are not prone to selection—showed few differences between these elements.

Another explanation for the low divergence of the rice and millet MULE sequences could be that they occur within a genomic region that, for whatever reason, experienced lower than average mutation rates. If this were the case, sequences adjacent to the elements should also show reduced variation. The authors tested this alternative hypothesis with the help of maize, which has more genomic sequence available than millet, by comparing genes flanking MULE regions in rice with evolutionarily conserved sequences in maize. The sequences did not show the similar degree of reduced variation predicted for below-average mutation rates.

Since neither selection nor low mutation frequency can explain the similar DNA between the grasses, the authors conclude, a transposon must have carried it between millet and rice long after these species diverged. Interestingly, the authors also found similar sequences in bamboo, raising the question of how common horizontal transfer may be between plant species. Given that plant mitochondrial genes appear “particularly prone to horizontal transfer,” the authors note, “it is remarkable that these results represent the first well-documented case of horizontal transfer of nuclear genes between plants.” But as researchers begin to explore the growing databases of plant genomic sequences, they can determine whether this finding constitutes an anomaly—or points to a significant force in plant genome evolution. *—Don Monroe*

